# Association of baseline, longitudinal serum high-sensitive C-reactive protein and its change with mortality in peritoneal dialysis patients

**DOI:** 10.1186/s12882-017-0624-4

**Published:** 2017-07-04

**Authors:** Wei Li, Liping Xiong, Li Fan, Yating Wang, Xuan Peng, Rong Rong, Yagui Qiu, Jiani Shen, Jianxiong Lin, Xueqing Yu, Haiping Mao

**Affiliations:** 1 Department of Nephrology, The First Affiliated Hospital, Sun Yat-sen University, Key Laboratory of Nephrology, Ministry of Health of China, 58 Zhongshan Road II, Guangzhou, 510080 China; 2 Department of Nephrology, The Sixth Affiliated Hospital, Sun Yat-sen University, Guangzhou, China

**Keywords:** All-cause mortality, Cardiovascular mortality, Continuous ambulatory peritoneal dialysis, Longitudinal hs-CRP, Change of hs-CRP

## Abstract

**Background:**

The prognostic values of baseline, longitudinal high-sensitivity C-reactive protein (hs-CRP) and its change over time on mortality in patients undergoing continuous ambulatory peritoneal dialysis (CAPD) remain uncertain.

**Methods:**

We retrospectively studied 1228 consecutive CAPD patients from 2007 to 2012, and followed up through December 2014. Cox regression models were performed to assess the association of hs-CRP on outcomes using serum hs-CRP levels as: (1) stratified by tertile of baseline or longitudinal hs-CRP levels; (2) baseline or longitudinal hs-CRP levels as continuous variables; and (3) categorized by tertile of slopes of hs-CRP change per year for each subject.

**Results:**

Higher baseline hs-CRP levels were not associated with clinical outcomes after adjustment for potential confounders. However, patients with the upper tertile of longitudinal hs-CRP had a nearly twice-fold increased risk of both all-cause and cardiovascular mortality [adjusted hazard ratio (HR) 1.77; (95% CI 1.16–2.70) and 2.08 (1.17–3.71), respectively], as compared with those with lower tertile. Results were similar when baseline or longitudinal hs-CRP was assessed as continuous variable. Additionally, the risk of all-cause and cardiovascular mortality in patients with increased trend in serum hs-CRP levels over time (tertile 3) was significantly higher [adjusted HR 2.48 (1.58–3.87) and 1.99 (1.11–3.56), respectively] when compared to those with relatively stable hs-CRP levels during follow-up period. These associations persisted after excluding subjects with less than 1-year follow up.

**Conclusions:**

Higher longitudinal serum hs-CRP levels and its elevated trend over time, but not baseline levels were predictive of worse prognosis among CAPD patients.

## Background

High-sensitivity C-reactive protein (hs-CRP) is used as a marker of systemic inflammatory in the clinical setting [1, 2]. Previous studies have demonstrated that an elevated serum hs-CRP level at a single time point is an important predictor of cardiovascular events both in general population [3, 4] and dialysis patients [5–11]. The relationship is seen even when controlling for usual established cardiovascular disease (CVD) risk factors.

However, hs-CRP levels are not static which may reflect a chronic inflammatory process in the dialysis patients, intercurrent clinical events, comorbidities, protein-energy wasting, decreased residual renal function and dialysis modality [12–15]. In a study of hemodialysis (HD) patients, serum CRP levels increased annually during 3 years of follow-up [16]. Further, HD patients with persistently high CRP levels but not an elevated CRP level only at a signal time point was associated with increased mortality risk, compared with a persistently low CRP levels [17, 18]. Additionally, biological inter-individual and intra-individual variability of CRP levels in dialysis patients might be greater than the variability in health individuals [1]. Nevertheless, only a few small studies have assessed the consequences of longitudinal conventional CRP, but not hs-CRP, fluctuation on mortality among peritoneal dialysis (PD) patients [19, 20].

Therefore, the relationship between longitudinal hs-CRP and its change and survival in PD patients has not been completely identified. In this retrospective cohort study, we sought to investigate the association of baseline, longitudinal hs-CRP levels and its change with all-cause and CVD mortality among continuous ambulatory peritoneal dialysis (CAPD) patients. We hypothesized that serial monitoring of hs-CRP levels could better capture prognostic information than a single baseline determination.

## Methods

### Subjects

In this retrospective cohort study, all consecutive CAPD patients were studied in our database between January 2007 and December 2012. Patients older than 18 years old undergoing CAPD treatment for more than 3 months were included. We excluded participants who had a history of HD (*n* = 59) or renal transplantation (*n* = 10) for more than 3 months, acute infection within 4 weeks of measurement (*n* = 22), malignancy (*n* = 24), or no baseline hs-CRP available (*n* = 87). At last, a total of 1228 patients were included in this study. Our study was approved by the First Affiliated Hospital of Sun Yat-sen University Institutional Review Boards. All subjects provided their written informed consent before enrollment.

### Data collection

All data used in this study were obtained from our database. Information collected at the time of PD initiation, including a complete history, physical examination, demographic characteristics, primary cause of end-stage renal disease, presence of diabetes and CVD. Presence of diabetes was defined as a self-reported history of physician diagnosis, the use of insulin or oral hypoglycemic agents, or a fasting glucose level of 126 mg/dL or greater. Presence of CVD was defined as congestive heart failure, ischemic heart disease, cerebrovascular disease, and peripheral vascular disease. Peritonitis was defined by the presence of at least two of the following criteria: (1) cloudy PD effluent; (2) white blood cell count in PD effluent more than 100/mm^3^ with 50% polymorphonuclear leukocytes; and (3) a positive culture from PD effluent [21].

All baseline biochemical parameters were obtained 3 months after PD initiation. To explore the changes in nutritional status and residual renal function during follow-up, we also included all available serum albumin, body mass index (BMI), and residual glomerular filtration rate (rGFR) data. Serum hs-CRP levels were regularly measured in our PD patients, and analyzed by an immunoturbidimetric assay (Beckman Coulter AU5800, USA) with a detection limit of 0.01 mg/L. Adequacy of dialysis assessed using total weekly urea clearance and total weekly creatinine clearance were calculated by using PD Adequest software 2.0 (Baxter Healthcare Corporation, Chicago, 1 L, USA). Residual renal function was estimated from mean values of urea clearance and creatinine clearance and adjusted for body surface area.

### Outcomes

The primary outcomes were all-cause and CVD mortality. CVD mortality was defined as death from myocardial infarction, heart failure, cerebrovascular accident, peripheral vascular accident and sudden death. Sudden death was diagnosed ad unexpected natural death occurring within 1 h of the onset of symptoms and without any prior condition that would appear fatal [22]. Survival time was defined as the time from enrollment to death or administrative censoring, including renal transplantation, transfer to HD or other dialysis centers, lost to follow-up, or end of the study period (December 31, 2014).

### Statistical analyses

Results were expressed as means ± standard deviation for normally distributed continuous variables, medians (interquartile ranges) for skewed distributed continuous variables and frequencies (%) for categorical variables. Comparisons between variables among different group of baseline hs-CRP level were performed using linear trend test, Kruskal-Wallis test or Chi-Square test, as appropriate. Survival curves of participants were generated by Kaplan-Meier method. Differences in the survival cures among four groups were compared by log-rank test.

Serum hs-CRP levels were used as: (1) stratified by tertile of baseline or longitudinal hs-CRP levels; (2) baseline or longitudinal hs-CRP levels as continuous variables; and (3) categorized by tertile of slope of hs-CRP change per year. Because of skewed distributions, the value of hs-CRP was log-transformed. The association between baseline serum hs-CRP levels and outcomes was assessed in Cox proportional hazards models. Hazard ratios (HRs) and 95% confidence intervals (95% CI) for each variable were calculated using a Cox proportional hazards model. Variables that demonstrated an unadjusted *P* value of <0.10 in univariate Cox proportional hazard regression analyses or for importance of clinical concern were included in the full model.

To incorporate longitudinal serum hs-CRP data and further calculate HRs for hs-CRP as time-dependent variable, extended Cox proportional hazards models were used [23, 24]. Briefly, we included all available hs-CRP data from the initiation of CAPD therapy to death, administrative censoring, or the end of study period, but not the measurements tested within 4 weeks of acute infection. To this end, 8298 hs-CRP measurements from 1228 subjects were included to examine the association between longitudinal hs-CRP levels and mortality. Furthermore, 8053 measurements from 983 patients with at least *2* hs-CRP data were used to investigate the association between hs-CRP change and mortality. Patients were grouped into three categories by the tertiles of slopes of hs-CRP change per year for each subject. Linear regression models were constructed for each patient to calculate least-squares estimation of slopes for assessing longitudinal within-subject change in serum hs-CRP, serum albumin, BMI and rGFR per year [25–27]. In sensitivity analyses, to minimize possible effects of reverse causality related to pre-existing disease, all hazard ratios were recalculated after excluding patients with follow-up periods less than 1 year.

All statistical analyses were performed using SPSS software, version 19.0 (SPSS Inc., Chicago, IL, USA), STATA, version 12.0 (stata, College Station, TX, USA.) and R (version 3.2.2; Free Software Foundation Inc., www.r-project.org).A *P* value <0.05 was considered statistically significant.

## Results

### Study participants

Baseline demographic and clinical characteristics of the cohort are presented in Table 1, stratified by baseline serum hs-CRP concentration tertiles. In total 1228 patients with a mean of 46.96 ± 14.9 years were enrolled in the study. Among them, 38.8% were female, 25.5% were diabetes, and 36.9% had a history of CVD. Median baseline serum hs-CRP level was 1.78 (0.66–5.79) mg/L. Patients in the upper tertile of baseline hs-CRP were older, were more likely to be male; had greater proportion of diabetes and pre-existing CVD; higher levels of body mass index, uric acid, triglyceride and low-density lipoprotein cholesterol; lower levels of mean arterial pressure, hemoglobin, serum albumin, pre-albumin and high-density lipoprotein cholesterol compared to those in the lower tertile (*P* < 0.05). During a median follow-up period of 35.0 (range 18.7 to 52.3) months, all-cause mortality was 19.5% (*n* = 240), in which CVD mortality was 57.5% (*n* = 138). In the entire cohort, the 653 episodes of PD-related peritonitis were identified in 377 (30.7%) patients during a cumulative follow-up period of 4111.9 patient-years, the overall peritonitis rate was 0.16 episodes per patient-year.

**Table 1:** Baseline characteristics of patients stratified by tertiles of baseline hs-CRP level

Variables	Baseline hs-CRP(mg/L)			*P* trend
	≤0.91 (*n* = 408)	0.92–3.74 (*n* = 410)	≥3.75 (*n* = 410)	
Age (year)	42.7 ± 14.0	46.3 ± 14.1	52.6 ± 15.1	<0.001
Female (%)	194 (47.5)	137 (33.4)	165 (40.2)	<0.001
BMI (kg/m^2^)	20.6 ± 2.8	21.9 ± 2.9	22.3 ± 3.5	<0.001
MAP (mmHg)	102.4 ± 13.5	103.1 ± 14.9	100.0 ± 14.6	0.018
Etiology of ESRD (%)				<0.001
Chronic glomerulonephritis	292 (71.6)	252 (61.5)	207 (50.5)	
Hypertensive nephropathy	20 (4.9)	29 (7.1)	39 (9.5)	
Diabetic nephropathy	70 (18.2)	94 (22.9)	113 (27.6)	
Other	26 (6.4)	35 (8.5)	51 (12.4)	
History of CVD (%)	138 (33.8)	153 (37.3)	192 (46.8)	<0.001
DM (%)	77 (18.9)	103 (25.1)	134 (32.7)	<0.001
HGB (g/L)	107.8 ± 21.7	109.8 ± 20.2	104.2 ± 20.3	0.013
ALB (g/L)	38.3 ± 4.5	37.8 ± 4.9	36.7 ± 5.1	<0.001
Prealbumin (mg/L)	385.5 ± 92.6	357.6 ± 92.0	299.6 ± 93.9	<0.001
UA (μmol/L)	403.3 ± 84.8	415.8 ± 83.3	429.2 ± 94.2	<0.001
Calcium (mmol/L)	2.3 ± 0.2	2.3 ± 0.2	2.3 ± 0.2	0.197
Phosphorus (mmol/L)	1.4 ± 0.4	1.4 ± 0.4	1.4 ± 0.4	0.968
iPTH (pg/mL)	212.9 (80.4, 410.9)	239.5 (116.4, 397.8)	203.2 (87.9, 386.7)	0.451
TCHOL (mmol/L)	4.9 (4.3, 5.7)	5.1 (4.3, 5.8)	5.0 (4.3, 5.9)	0.104
TG (mmol/L)	1.4 (1.0, 1.8)	1.4 (1.0, 1.9)	1.7 (1.1, 2.5)	<0.001
HDL-C (mmol/L)	1.3 (1.1, 1.6)	1.2 (1.0, 1.5)	1.1 (0.9, 1.4)	<0.001
LDL-C (mmol/L)	2.8 (2.3, 3.4)	3.0 (2.4, 3.6)	2.9 (2.3, 3.6)	0.049
rGFR (ml/min/1.73 m^2^)	3.7 ± 3.1	3.9 ± 2.6	3.8 ± 2.8	0.658

*Abbreviations*: *BMI* body mass index; *MAP* mean arterial pressure; *ESRD* end stage renal disease; *CVD* cardiovascular disease;

*DM* diabetes mellitus; *HGB* hemoglobin; *ALB* serum albumin; *UA* uric acid; *iPTH* parathyroid hormone; *TCHOL* total cholesterol;

*TG* triglyceride; *HDL-C* high-density lipoprotein cholesterol; *LDL-C* low-density lipoprotein cholesterol; *rGFR* residual glomerular filtration rate.

### Baseline hs-CRP levels and mortality

**Fig. 1:**
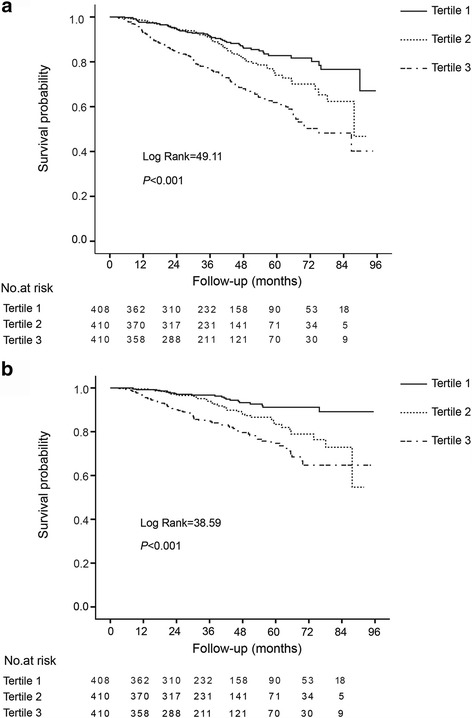
Kaplan-Meier survival curves for all-cause mortality (**a**) and CVD mortality (**b**) according to tertiles of hs-CRP levels at baseline. The *P* values refer to the significance of the log-rank test across quartiles

Kaplan-Meier survival curves for all-cause and CVD mortality by quartiles of baseline hs-CRP levels are shown in Fig. 1. Patients in the upper tertiles (tertile 3) had the worst all-cause and cardiovascular survival rates among the groups (*P* < 0.001). However, after adjustment for potential confounding factors, the upper hs-CRP tertile was not predictive of all-cause and CVD mortality as compared with the lower tertile (tertile 1). When treating hs-CRP as a continuous variable after log transformation, baseline hs-CRP level was not associated with all-cause mortality [HR = 1.19 (95% CI 0.89–1.60); *P* = 0.246], but borderline significantly associated with CVD mortality [HR = 1.50 (95% CI 1.00–2.24); *P* = 0.048] (Table 2). In sensitivity analysis, we excluded patients with less than 1-year follow up, the results were materially unchanged (data not shown).

**Table 2:** Associations of baseline hs-CRP and adjusted variables^a^ with all-cause and CVD mortality

	All-cause mortality		CVD mortality	
	HR (95% CI)	*P* value	HR (95% CI)	*P* value
hs-CRP				
Log hs-CRP(per 1 mmol/L)	1.19 (0.89–1.60)	0.246	1.50 (1.00–2.24)	0.048
hs-CRP tertile 1	Ref.	-	Ref.	-
hs-CRP tertile 2	1.09 (0.71–1.66)	0.692	1.39 (0.77–2.53)	0.273
hs-CRP tertile 3	1.26 (0.83–1.90)	0.273	1.63 (0.90–2.93)	0.104
Age (per 1 year)	1.04 (1.03–1.06)	<0.001	1.04 (1.02–1.06)	<0.001
Sex (female versus male)	0.85 (0.63–1.16)	0.304	0.79 (0.53–1.18)	0.248
BMI (per 1 kg/m^2^)	1.02 (0.96–1.08)	0.485	1.08(1.01–1.16)	0.025
MBP (per 10 mmHg)	1.03 (0.94–1.14)	0.497	1.03 (0.91–1.17)	0.591
CVD (yes versus no)	2.43 (1.77–3.34)	<0.001	3.35 (2.15–5.21)	<0.001
DM (yes versus no)	2.03 (1.45–2.85)	<0.001	1.94 (1.26–3.01)	0.003
HGB (per 10 g/L)	0.92 (0.85–1.01)	0.070	0.94 (0.84–1.05)	0.237
ALB (per 1 g/L)	0.94 (0.91–0.98)	0.003	0.94 (0.90–0.99)	0.023
UA (per 10 μmol/L)	1.01 (0.99–1.03)	0.257	1.00 (0.98–1.03)	0.844
TG (per 1 mmol/L)	1.12 (1.02–1.24)	0.021	1.13 (1.00–1.28)	0.054
rGFR (per 1 ml/min/1.73 m^2^)	0.94 (0.89–1.00)	0.058	0.92 (0.85–1.00)	0.043

^a^ Variable that demonstrated an unadjusted *P* value of <0.10 in univariate Cox proportional hazard regression analyses or for importance of clinical concern was included in the this model.

*Abbreviations*: *BMI* body mass index; *MAP* mean arterial pressure; *CVD* cardiovascular disease; *DM* diabetes mellitus; *HGB* hemoglobin;

*ALB* serum albumin; *UA* uric acid; *TG* triglyceride; *rGFR* residual glomerular filtration rate.

### Longitudinal hs-CRP levels and mortality

Overall median longitudinal hs-CRP concentration was 2.08 (0.78 to 6.47) mg/L, and median series of serum hs-CRP were 5 times per patient (interquartile range 2–10 times) with follow-up up to median of 35 months. Table 3 presents results of extended Cox proportional hazards models for the relationship between longitudinal measurements of hs-CRP tertile and mortality. The adjusted all-cause and CVD mortality HRs for the upper tertile (tertile 3) were 1.77 (95% CI 1.16–2.70; *P* = 0.008) and 2.08 (95% CI 1.17–3.71; *P* = 0.013), respectively as compared with the lower tertile of hs-CRP (tertile 1) (P for trend < 0.05). Similar results were obtained when longitudinal hs-CRP was included as a continuous variable. Each 1 mg/L elevation in log longitudinal hs-CRP levels associated with a 64% increase in all-cause (95% CI 1.21–2.22; *P* = 0.002) and 92% increase in CVD death risk (95% CI 1.28–2.88; *P* = 0.002). Consistently, the estimates were robust in sensitivity analysis (data not shown).

**Table 3:** Associations of longitudinal hs-CRP with all-cause and CVD mortality

	Model 1^a^		Model 2^b^		Model 3^c^	
	HR(95% CI)	*P* value	HR(95% CI)	*P* value	HR (95% CI)	*P* value
All-cause mortality						
Log hs-CRP (per 1 mg/L)	2.78 (2.16–3.56)	<0.001	1.76 (1.36–2.29)	<0.001	1.64 (1.21–2.22)	0.001
Tertile 1	Ref.	-	Ref.	-	Ref.	-
Tertile 2	1.92 (1.31–2.80)	<0.001	1.44 (0.98–2.11)	0.061	1.42 (0.91–2.20)	0.118
Tertile 3	3.38 (2.38–4.80)	<0.001	1.90 (1.32–2.72)	0.001	1.77 (1.16–2.70)	0.008
*p* trend	<0.001		<0.001		0.007	
CVD mortality						
Log hs-CRP (per 1 mg/L)	3.27 (2.33–4.59)	<0.001	1.98 (1.39–2.83)	<0.001	1.92 (1.28–2.88)	0.002
Tertile 1	Ref.	-	Ref.	-	Ref.	-
Tertile 2	2.32 (1.37–3.94)	0.002	1.70 (0.99–2.90)	0.053	1.58 (0.86–2.88)	0.139
Tertile 3	4.25 (2.59–6.96)	<0.001	2.24 (1.35–3.72)	0.002	2.08 (1.17–3.71)	0.013
*p* trend	<0.001		0.002		0.010	

*HR* hazard ratio, *95% CI* 95% confidence interval.

^a^Model 1: unadjusted.

^b^Model 2: adjusted for age, sex, CVD, DM.

^c^Model 3: model 2 adjusted for BMI, MAP, hemoglobin, albumin, triglyceride, uric acid, rGFR and peritonitis event occurred during follow-up period.

### Hs-CRP change and mortality

**Fig. 2:**
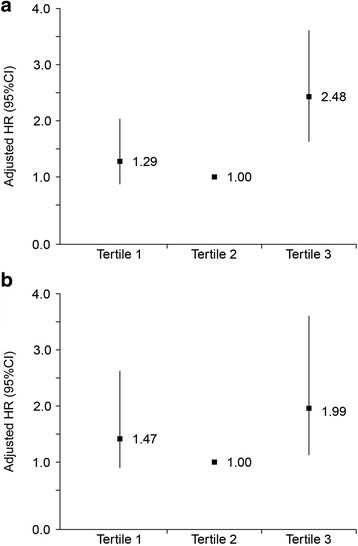
Hazard ratio for all-cause (**a**) and CVD mortality (**b**) according to tertile of slope of hs-CRP change per year

Further analyses of the association between serum hs-CPR and mortality were performed after stratifying by the tertile of slope of hs-CPR change per year. Among 983 patients with at least 2 hs-CRP data, 334 (34.0%) patients presented total 589 episodes of peritonitis during a cumulative follow-up period of 3601.3 patient-years. The overall peritonitis rate was 0.16 episode per patient-year. As shown in Table 4, there are no significantly differences in baseline characteristics among groups. During follow-up, nutritional status, as reflected by changes in BMI and serum albumin levels, and residual renal function, as reflected by changes in residual glomerular filtration rate (rGFR) varied over time. Across all three groups, the rGFR of patients in tertile 3 declined faster compared with other groups, whereas there was no difference in nutritional status changes among three groups (Table 5). The relative risk of mortality in relation to hs-CRP change showed in Table 6 and Fig. 2, with subjects in the relatively stable hs-CRP levels over time (tertile 2) as a reference. Patients with increased trend in hs-CRP levels (tertile 3) were at elevated risk of both all-cause and CVD mortality, with adjusted HRs of 2.48 (1.58–3.87) and 1.99 (1.11–3.56), respectively, independently of the baseline serum hs-CRP levels. Furthermore, the results remained significantly after adjusting for rGFR change and PD-related peritonitis event during follow-up. Finally, the sensitivity analysis indicated the same findings after excluding patients with less than 1-year follow-up period (data not shown).

**Table 4:** Baseline characteristics of patients stratified by tertiles of change of hs-CRP

Variables	Change of hs-CRP (mg/L per year)^a^			*P* trend
	Tertile 1 ≦ −0.03 (*n* = 327)	Tertile 2 −0.02-0.12 (*n* = 328)	Tertile 3 ≧0.13 (*n* = 328)	
Baseline hs-CRP (mg/L)	3.7 (1.3,9.4)	2.1(0.7,8.3)	0.9 (0.4,2.2)	<0.001
Age (year)	46.8 ± 14.7	48.8 ± 14.8	46.8 ± 15.0	0.994
Female (%)	129(39.4)	128(39.0)	143(43.6)	0.420
BMI (kg/m^2^)	21.7 ± 3.1	21.7 ± 3.1	21. ± 3.1	0.980
MAP (mmHg)	101.2 ± 15.4	100.8 ± 14.0	103.0 ± 14.3	0.124
Etiology of ESRD (%)				0.464
Chronic glomerulonephritis	202(61.8)	189(57.6)	203(61.9)	
Hypertensive nephropathy	25(7.6)	26(7.9)	24(7.3)	
Diabetic nephropathy	67(20.5)	83(25.3)	77(23.5)	
Other	33(10.1)	30(9.1)	24(7.3)	
History of CVD (%)	133(40.7)	126(38.4)	129(39.3)	0.838
DM (%)	78(23.9)	92(28.0)	87(26.5)	0.466
HGB (g/L)	107.1 ± 19.0	109.6 ± 20.0	109.5 ± 20.4	0.120
ALB (g/L)	37.7 ± 4.6	38.4 ± 4.5	37.7 ± 4.6	0.988
Prealbumin (mg/L)	335.7 ± 97.9	346.6 ± 101.8	360.7 ± 93.5	0.001
UA (μmol/L)	422.1 ± 91.1	420.6 ± 86.1	404.6 ± 82.5	0.010
Calcium (mmol/L)	2.3 ± 0.2	2.3 ± 0.2	2.3 ± 0.2	0.511
Phosphorus (mmol/L)	1.4 ± 0.4	1.4 ± 0.4	1.4 ± 0.4	0.950
iPTH (pg/mL)	214.6(106.3391.3)	202.4 (84.7389.1)	243.3(99.6407.2)	0.426
TCHOL (mmol/L)	5.0 (4.1,5.8)	5.1 (4.4,5.9)	5.2(4.4,5.9)	0.333
TG (mmol/L)	1.5(1.0,2.1)	1.5(1.1,2.1)	1.4(1.0,2.0)	0.538
HDL-C (mmol/L)	1.2(0.9,1.4)	1.2 (1.0,1.5)	1.2(1.0,1.5)	0.004
LDL-C (mmol/L)	2.9 (2.3,3.5)	3.0(2.4,3.6)	2.9(2.4,3.5)	0.907
rGFR (ml/min/1.73 m^2^)	3.6 ± 2.6	4.2 ± 3.3	3.7 ± 2.6	0.559

^a^The value of hs-CRP was log-transformed.

*Abbreviations*: *BMI* body mass index; *MAP* mean arterial pressure; *ESRD* end stage renal disease; *CVD* cardiovascular disease;

*DM* diabetes mellitus; *HGB* hemoglobin; *ALB* serum albumin; *UA* uric acid; *iPTH* parathyroid hormone; *TCHOL* total cholesterol;

*TG* triglyceride; *HDL-C* high-density lipoprotein cholesterol; *LDL-C* low-density lipoprotein cholesterol; *rGFR* residual glomerular filtration rate.

**Table 5:** Change in nutritional parameters and residual glomerular filtration rate among patients stratified by change of hs-CRP

Variables	Change of hs-CRP (mg/L per year)^a^			*P* trend
	Tertile 1 (*n* = 327)	Tertile 2 (*n* = 328)	Tertile 3 (*n* = 328)	
Median (IQR) BMI change, kg/m^2^ per year	0.33(−0.14,1.00)	0.34(−0.04,0.76)	0.54(−0.03,1.21)	0.682
Median (IQR) ALB change, g/L per year	−0.57(−2.25,1.02)	−0.61(−1.43,0.08)	−0.82(−2.53,0.65)	0.077
Median (IQR) rGFR change, ml/min/1.73 m^2^per year	−1.04(−1.76,-0.45)	−0.89(−1.73,-0.46)	−1.12(−2.40,-0.44)	0.004

^a^The value of hs-CRP was log-transformed.

*Abbreviations*: *BMI* body mass index; *ALB* serum albumin; *rGFR* residual glomerular filtration rate.

**Table 6:** Associations of change in hs-CRP with all-cause and CVD mortality

	All-cause mortality		CVD mortality	
	HR(95% CI)	*P* value	HR(95% CI)	*P* value
Unadjusted model				
tertile 1 versus tertile 2	1.37 (0.95–1.97)	0.092	1.41 (0.90–2.21)	0.131
tertile 3 versus tertile 2	1.68 (1.19–2.37)	0.003	1.41 (0.90–2.20)	0.131
General adjusted model^a^				
tertile 1 versus tertile 2	1.33 (0.88–2.01)	0.179	1.36 (0.81–2.26)	0.244
tertile 3 versus tertile 2	1.93 (1.32–2.81)	0.001	1.55 (0.94–2.56)	0.084
General adjusted model + baseline hs-CRP level				
tertile 1 versus tertile 2	1.25 (0.82–1.90)	0.292	1.27 (0.76–2.12)	0.358
tertile 3 versus tertile 2	2.38 (1.58–3.57)	<0.001	1.98 (1.16–3.38)	0.012
General adjusted model + baseline hs-CRP level + rGFR change				
tertile 1 versus tertile 2	1.29 (0.81–2.08)	0.286	1.47(0.82–2.62)	0.193
tertile 3 versus tertile 2	2.48(1.58–3.87)	<0.001	1.99(1.11–3.56)	0.021

^a^General adjusted model: adjusted for age, sex, CVD, DM, BMI, MAP, hemoglobin, albumin, TG, uric acid, rGFR and peritonitis event occurred during follow-up period.

## Discussion

In the present study, we found that higher baseline serum hs-CRP levels were not associated with elevated risk of all-cause and CVD mortality in CAPD patients. However, higher longitudinal hs-CRP levels over time were predictive of both all-cause and CVD mortality. We also observed that an increasing trend in hs-CRP level, independently of baseline hs-CRP levels and rGFR change during follow-up, significantly increases both risk of all-cause and CVD mortality in CAPD patients. These results were confirmed in sensitivity analyses after excluding subjects with follow up of less than 1-year.

Although there generally was agreement between baseline serum CRP levels with outcomes in PD patients, discordant findings were observed across studies [8–11, 19, 28]. In a prospective study of 50 PD patients from a single center in Australia [28], an elevated CRP level was independently associated with CVD events, but not with all-cause mortality. However, a single prospective cohort consisting of 246 incident CAPD patients showed a greater risk of all-cause and CVD mortality among patients with higher hs-CRP levels [9]. Another study including 402 Taiwanese CAPD patients found that every 1 mg/L increase in hs-CRP level was independently predictive of a 1.4% increase in all-cause mortality [11]. In our study, we did not found the independent relationship between baseline serum hs-CRP and all-cause or CVD mortality. However, higher serum albumin level was associated with decreased mortality risk after multivariate adjustment in our present study. Since serum albumin is not only the marker of nutrition, but also a negative acute-phase protein with longer half-time compared with CRP, this may be partly explained that why baseline hs-CRP loses its ability to predict long-term outcomes after adjusted for serum albumin.

Owing to the pretty short half-life of CRP and change in clinical status, the association between baseline CRP and all-cause mortality may alter over prolonged follow-up periods. Indeed, CRP level was noted to vary with time on PD [19, 29]. However, current evidence regarding the relationship and mortality is mainly derived from cross-sectional studies, limited data is available about the association of longitudinal CRP and its change with outcomes in dialysis patients [19, 20]. A prospective study included 97 PD patients with 5 serum CRP measurements collected every 4 months and showed the averaged value of CRP was more predictive of prognosis compared to the baseline value [19]. In the Netherlands Cooperative Study on the Adequacy of Dialysis (NECOSAD), including 635 participants from 38 Dutch dialysis centers with 2 CRP measurements at 3 and 6 months of follow up, patients with repeatedly high concentrations of CRP were positively related to risk of cardiovascular events and mortality [20]. Notably, both studies included relatively few longitudinal measurements and measured CRP concentration by using a conventional detection method with a low sensitivity. Our study included more than 8000 serial hs-CRP data with longer follow-up (35 months) and we also found that higher longitudinal hs-CRP levels and its change were associated with poor prognosis. Intriguingly, we observed that patients, even with low baseline hs-CRP concentrations, but exhibiting increased hs-CRP trend, had higher risk of all-cause and CVD mortality. Such associations remain robust even after multivariate adjustment, including peritonitis event occurred during follow-up period. These results indicate that the persistent state of nonspecific micro-inflammation may contribute to the poor outcome of PD patients. Previous studies have report that loss of residual renal function was associated with higher levels of CRP, IL-6 and other inflammatory markers, suggesting that decreased residual kidney function per se may contribute to the development of the inflammatory milieu, directly or indirectly [15, 30]. Thus, patients in increased hs-CRP group may be due partly to faster decline of residual renal function in our study. Further, we excluded patients with follow-up less than 1-year, the results did not substantially alter. These findings indicate that hs-CRP is the predictor of risk in the short term and serial measurements in clinical practice may add significant prognostic value to a baseline measurement among PD patients.

The mechanisms by which hs-CRP affects the risk of developing future CVD events involve various factors. One explanation has been provided that systemic inflammation play a key role in the pathogenesis of atherosclerosis [31]. Although previous studies have been demonstrated a graded, dose-response relationship between hs-CRP levels and risk of coronary disease [32, 33], peripheral arterial disease [34, 35] and sudden death [36], it is unknown whether or not elevated CRP levels directly contribute to the development of CVD. Some studies revealed the prothrombotic effects of CRP on human endothelial cells, partially via inhibiting tissue plasminogen activator expression and activity, or reducing prostacyclin release [37–39]. In contrast, data from the Mendelian randomization genetic studies suggested that the CRP more likely is an innocent bystander than a cause of atherosclerosis [40, 41]. Nevertheless, future studies are required to elucidate the underlying mechanisms.

The major strength of this study is the relatively large number of included longitudinal hs-CRP measurements in CAPD patients during follow-up. Our study also has several limitations. First, it was a single-center study, and a center-specific effect cannot be excluded. Secord, there is a potential selection bias when we evaluated the association between hs-CRP change and mortality. Because we included patients with at least two check-ups of hs-CRP, patients with relatively higher hs-CRP levels might die until next measurement texted. Third, given the retrospective nature of the cohort, we could not completely exclude all measurements of hs-CRP during acute infection events occurrence, as well as the possible of unmeasured confounding.

## Conclusion

Higher baseline serum hs-CRP levels were not associated with elevated risk of all-cause and CVD mortality in CAPD patients. However, higher longitudinal and increasing trend of hs-CRP levels over time are positively related to all-cause and CVD mortality. This association of hs-CRP change and poor prognosis was independently baseline levels of hs-CRP.

## Abbreviations

ALB: Serum albumin
BMI: Body mass index
CAPD: Continuous ambulatory peritoneal dialysis
CI: 95% confidence intervals
CVD: Cardiovascular disease
DM: Diabetes mellitus
ESRD: End stage renal disease
HD: Hemodialysis
HDL-C: High-density lipoprotein cholesterol
HGB: Hemoglobin
HR: Hazard ratio
hs-CRP: High-sensitivity C-reactive protein
iPTH: Parathyroid hormone
LDL-C: Low-density lipoprotein cholesterol
MAP: Mean arterial pressure
PD: Peritoneal dialysis
rGFR: Residual glomerular filtration rate
TCHOL: Total cholesterol
TG: Triglyceride
UA: Uric acid
